# Association between Ambient Illumination and Cognitive Impairment: A Population-Based Study of Older

**DOI:** 10.1155/2023/4131377

**Published:** 2023-04-10

**Authors:** Tieyi Shi, Baozhong Chen

**Affiliations:** Heilongjiang University of Chinese Medicine, Harbin, China

## Abstract

It is well-established that light therapy can alleviate cognitive impairment, and ambient illumination (AI) can quantify the amount of exposure to light. However, the relationship between AI and cognitive impairment has been largely understudied. *Objectives*. We aimed to examine the cross-sectional associations between AI and impaired cognition using data from the National Health and Nutrition Examination Survey (NHANES) (2011–2013) database. *Methods*. The correlation between AI and cognitive impairment was analyzed using multivariate logistic regression models. Nonlinear correlations were explored using curve fitting. *Results*. Multivariate logistic regression yielded an OR of 0.872 (95% CI 0.699, 1.088) for the association between AI and cognitive impairment after adjusting for covariates. Smooth curve fitting showed that the correlation was nonlinear, with an inflection point at 1.22. *Conclusions*. These results suggested that the level of AI may be linked to cognitive impairment. We found a nonlinear relationship of AI with cognitive impairment.

## 1. Background

Current evidence suggests that cognition decreases with aging and is affected by multiple factors, such as sex and education [1]. Impaired cognition is common in the elderly [2], and it has been reported that mild cognitive impairment may increase the risk of falls in this population [3]. Besides, severe cognitive impairment can lead to dementia and affect interpersonal communication in older adulthood [4, 5]. These complications raise the need for extra healthcare and severely affect the quality of life of the elderly.

It is well-established that comorbidities, including chronic kidney disease (CKD), hypertension, and metabolic syndrome (MetS), are common in older adults and can affect their cognitive function [6–8]. An increasing body of evidence suggests that renal insufficiency could induce neurotoxicity, hemodynamic changes, and cognitive decline [9–12]. It is now understood that hypertension damages cerebral blood vessels through oxidative stress and inflammation, causing arterioles in the brain to harden and bleed, and causing arterioles in the brain to harden and reshape, thus affecting cognitive function [13]. MetS can increase blood–brain barrier permeability, cause neuroinflammation, and increase cognitive impairment [14].

There is a growing consensus that light alleviates cognitive impairment [15, 16]. A previous study showed that emotional symptoms were reduced in older adults after 16 weeks of light therapy compared with the control group [17]. Another study showed that exposure to bright light (5,000 Lx) reduces sleepiness and fatigue compared to low light (10 Lx) [18]. Besides, in controlled experiments with ambient light, brighter lighting in classrooms improved math and reading performance, and blue-enriched white light could improve self-reported alertness, performance, and sleep quality [19, 20].

Given that cognitive performance is measured differently in older adults than in adolescents and adults, and in the present study, we sought to understand whether ambient light affects cognitive function in the elderly. Importantly, we explored the relationship between the intensity of ambient light exposure in the elderly's daily life and the degree of cognitive impairment.

## 2. Method

### 2.1. Study Population

The National Health and Nutrition Examination Survey (NHANES) is a cross-sectional survey that applies multiphase stratified sampling to analyze a representative sample of the US population. The survey protocol was approved by the National Center for Health Statistics Research Ethics Review Board. Written informed consent was obtained from all NHANES participants. NHANES staff first interviewed participants in their homes. Information about demographics and health status is collected during this time. Participants were instructed to perform physical examinations and laboratory testing in mobile examination centers (MEC).

**Figure 1:**
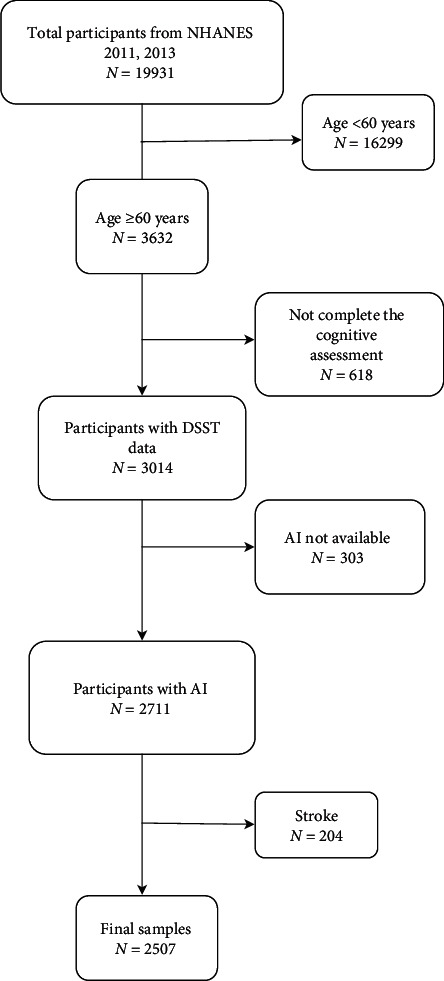
Flowchart of participant selection. NHANES, National Healthand Nutrition Examination Survey; DSST, digit symbol substitution test; AI, ambient illumination.

3,632 individuals over the age of 60 years were included in this study who participated in the continuous NHANES survey cycles of 2011–2012 through 2013–2014. After excluding 1,125 individuals with a history of stroke (*n* = 204), without information available on ambient illumination (AI) (*n* = 303) and who did not complete the cognitive assessment (*n* = 618), 2,507 individuals were enrolled in this study (Figure 1).

### 2.2. Ambient Illumination

Participants were asked to wear the physical activity monitor (PAM) day and night for 7 days, starting on the day of the test of MEC. On the morning of day 9, participants removed it. The device used in NHANES is the ActiGraph model GT3X+, which can measure ambient light levels every second [21]. We calculated the mean of ambient light (lux) at the minute level and then added the mean to obtain the daily data. Indeed, ambient light levels can reflect the amount of light the wearer is exposed to in daily life. We averaged the 9-day data and divided it by 100,000.

### 2.3. Assessment of Cognitive Function

The digit symbol substitution test (DSST) was used in the present study to assess cognitive function. Participants were asked to draw as many symbols paired with numbers as possible within 2 minutes. A score was given for each correctly drawn symbol according to the standard scoring method, with higher scores indicating better cognitive function. We defined scores in the study group below the lowest quartile (≤34 points) as indicating cognitive impairment, with the rest considered normal [22].

Further, the susceptibility tests were conducted three times using word learning and recall modules from the Consortium to Establish a Registry for Alzheimer's Disease (CERAD-Word list memory test (WL)), the CERAD delayed recall (CERAD-DR), and the Animal Fluency Test (AFT) separately. CERAD-WL consists of three consecutive tests, and CERAD-DR was performed about 8–10 minutes after CERAD-WL. Participants were instructed to read aloud and memorize 10 unrelated words in the learning test. In each test, the order of the 10 words changes. The highest score per test is 10 points. During the AFT, participants were asked to name as many animals as possible in a minute. 1 point is awarded for each animal name. Based on the literature, the cut-off values for cognitive impairment are: <17 for CERAD-WL, <5 for CERAD-DR, and <14 for AFT [23, 24]. One score in the “impaired” range was sufficient to include a participant within the “cognitive impairment” group.

### 2.4. Covariates

Since CKD, hypertension, and metabolic disease are widely acknowledged to impact cognitive function, we use them as covariates. Urinary creatinine was assessed using the Roche/Hitachi Modular P Chemistry Analyzer, and a solid-phase fluorescent immunoassay was used to measure human urinary albumin. Urinary albumin/creatinine ratio (ACR) refers to the urine protein/creatinine ratio. estimated glomerular filtration rate (eGFR) was calculated using the chronic kidney disease epidemiology collaboration equation. The diagnostic criteria used for CKD were ACR > 30 or eGFR < 60 [25].

The measurements of waist circumference and blood pressure (systolic and diastolic blood pressure) were performed at the MEC. The DxC800 uses a timed-endpoint method to determine the concentration of triglycerides (TG) in serum or plasma and glucose oxidase method to glucose [26]. High-density lipoprotein (HDL) formed a purple/blue pigment after adding multiple reagents to serum and was measured by photometry at 600 nm (secondary wavelength = 700 nm) [27].

Monitor-Independent Movement Summary (MIMS) triaxial value is the sum of the acceleration measurements obtained on all three axes at the level (the triaxial values for the *x*, *y*, and *z* axes). We summed the 9-day data and divided it by 1,000.

Hypertension was judged on questionnaires and blood pressure measurements. The questionnaire includes questions such as: Has your doctor ever told you that you have high blood pressure and whether you are taking high blood pressure medication? [28]. The cut-off values for hypertension are systolic blood pressure > 140 or diastolic blood pressure > 90. When three measurements of participants met the above conditions, they were diagnosed with hypertension [29]. The diagnostic criteria for MetS based on the Adult Treatment Panel III (ATP III) (2001) included any three of the following five features: waist circumference > 102 cm in men or >88 cm in women; lipid: TG > 150 mg/dL, or HDL < 40 mg/dL in men or <50 mg/dL in women; blood pressure: >130/85 mm Hg; glucose > 110 mg/dL (includes diabetes).

### 2.5. Statistical Analyses

A statistical weighting method was used to analyze our survey data. Descriptive statistics were used to depict the characteristics of the study participants. Continuous variables were expressed as weighted means and standard errors of the means. Categorical variables were expressed as numbers and frequencies.

When comparing descriptive statistics, weighted linear regression and chi-square tests were used for continuous and categorical variables. We used multiple regression analysis to assess the relationship between AI and cognitive impairment, built rough models, and conducted trend tests. We assessed the interaction effects between the lux value and MIMS triaxial value.

We performed smooth curve fitting after adjusting the variables. Further linear and piecewise linear models were performed to quantify the associations. The value with the highest likelihood was selected to build a two-line piecewise linear model if there was evidence of a nonlinear relationship. We performed the logistic regression analysis in two stages. Subgroup analyses were conducted to explore potential confounders.

## 3. Results

### 3.1. Characteristics of the Study Population

As shown in Table 1, the mean age of the participants was 69.01 ± 0.22 years, predominantly females (51.14%), and Caucasians (47.63%). Most participants had high school or higher levels of education (*n* = 1,303, 52.03%). A significant correlation was found between education level and cognitive impairment (*P* < 0.0001), with higher education level associated with lower prevalence of cognitive impairment. The mean AI was lower in participants with cognitive impairment than participants with normal cognition (*P* = 0.003). Besides, the prevalence of cognitive impairment in CKD and hypertensive populations was higher (Table 1).

### 3.2. Association between AI and Cognitive Impairment

**Figure 2:**
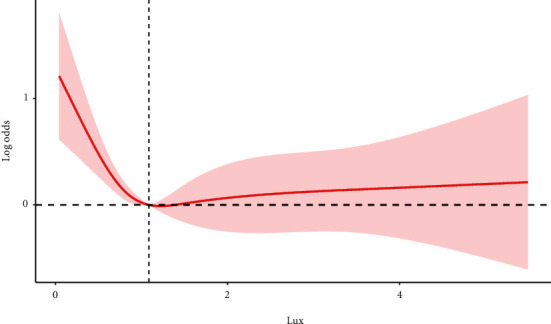
Association between AI and cognitive impairment. Age, gender, race, the education level, and diagnosis of CKD, MetS, and hypertension were adjusted (*P* for nonlinearity: 0.0005).

**Figure 3:**
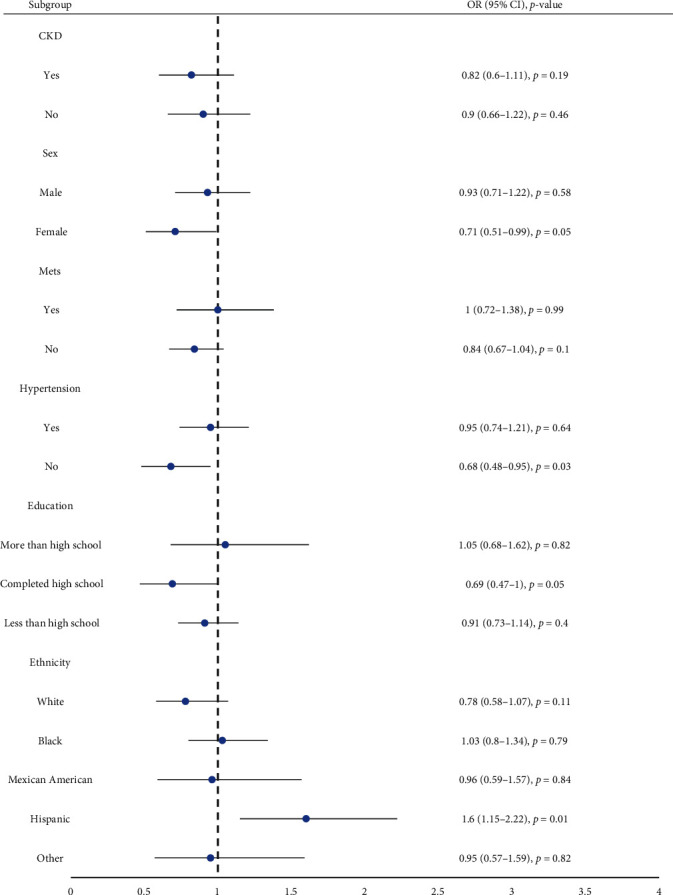
Subgroup analysis of risk factors for the relationship between AI and cognitive impairment. In the subgroup analysis stratified by age, gender, race, the education level, and diagnosis of CKD, MetS, and hypertension, the model is not adjusted for sex, race, education level, CKD, hypertension, and MetS, respectively.

Three models were constructed to examine the relationship between AI and cognitive impairment in this study. No covariate was adjusted for the crude model, while for Model 1, adjustments were made for age, gender, and race. Model 2 was similar to Model 1 except that the education level was added, and comorbidities such as CKD, MetS, and hypertension were used as covariates. In the crude model, the OR was 0.775 (95% CI 0.647, 0.927), which suggests that each increment in AI was associated with a 22.5% lower risk of cognitive impairment. For Model 1, the risk of cognitive impairment decreased by 11.8% for each increment in AI [0.882 (0.723, 1.077)] (Model 1). In the fully adjusted model (Model 2), the risk of cognitive impairment was reduced by 12.8% for each increment in AI [0.872 (0.699, 1.088)] (Table 2). However, there was no significant association between AI and cognitive impairment in the two adjusted groups. We found no interaction effects during regression analysis between the lux value and MIMS triaxial value [1.01 (0.97, 1.06)]. Consistent findings were obtained during sensitivity analysis, where AI was transformed into quartiles (Table 2). Next, we performed sensitivity analyses with CERAD-WL, CERAD-DR, and AFT (Supplemental Table 1). The estimated associations between AI (as a continuous variable) and cognitive impairment were visualized in a nonlinear spline model (Figure 2) and linear and piecewise linear models (Table 3). After adjusting for sex, age, race, education level, and comorbidities, including CKD, MetS, and hypertension (*P* for nonlinearity: 0.0005), the model computed an inflection point at 1.22. A negative correlation was found with cognitive impairment at AI values below 1.22 while no significant association was found at higher AI values. We explored the potential association between AI and the risk of cognitive impairment in different populations using subgroup analyses, stratifying according to sex, race, education level, CKD, MetS, and hypertension. Overall, our results suggest that in women and populations without hypertension, increasing AI could significantly reduce the incidence of cognitive impairment (Figure 3).

## 4. Discussion

In the present study, we found a negative correlation between AI and cognitive impairment in the crude model, suggesting that AI may be a protective factor against cognitive decline. Interestingly, with an increase in AI, the risk of cognitive impairment decreased. However, in the fully adjusted model, no significant association was observed. Restricted cubic spline regression showed that the negative correlation was nonlinear, with an infection point at an AI of 1.22. At AI values less than 1.22, there was a negative correlation between AI and cognitive impairment after adjusting all covariates. These results suggest that adequate ambient light exposure may slow cognitive decline in older adults, and an optimal benefit may be achieved when light exposure reaches the inflection point.

In recent years, ambient lighting-based studies have revealed that high illuminance environments could significantly promote subjective and objective alertness [30], sustained attention [31, 32], executive control [33], and working memory task performance [34]. These changes may be due to the impact of light on the body's circadian rhythm. Intriguingly, it has been reported that the cortisol arousal response (CAR) also affects cognitive function. CAR can predict executive performance, emotional response, and emotional regulation during the day [35]. In a comparative experiment, two groups of adolescents were exposed to different light intensities for 80 minutes after waking up, and CAR was assessed via their saliva samples. Importantly, it was found that exposure to bright light in the morning could significantly enhance CAR compared to dim light [36]. A decrease in CAR levels within 30 minutes of waking up in the morning can affect cognitive function [35], suggesting that ambient luminosity may affect cognitive function through CAR. These studies overlap in their collective assertion that both interventional phototherapy and ambient light positively affect cognitive function. Experimental studies showed that light stimulation of rod nerve and cone cells could promote the secretion of melatonin from the pineal gland through light-sensitive nerve cells, thus affecting the human circadian rhythm [37]. The effects of light on cognitive ability can be detected in multiple brain regions. It has been shown that the wavelength, duration, and intensity of light exposure could regulate alert-related subcortical structures and amygdala and hippocampus, adjust cortical areas and ultimately affect behaviors [38]. Ambient light also affects genes by activating the pituitary adenylate cyclase-activating polypeptide-extracellular signal-regulated kinase/mitogen-activated protein (PACAP-ERK/MAP) kinase signaling pathway and inducing the expression of genes associated with the circadian clock [39]. Moreover, enhanced lighting can increase serum vitamin D levels, while elevated serum vitamin D levels improve cognitive function [40].

It is well-established that older adults often exhibit reduced outdoor activities due to physical reasons and reduced social interaction. In this case, reduced ambient light stimulation may exacerbate the cognitive decline in older adults. The device used in NHANES to measure exposure to ambient light in the elderly population was ActiGraph model GT3X. This device measures ambient light intensity (lux) every 1 second (1 Hz). Although this is a crude measure, it allows the study to have more subjects. Ultimately, this study aims to identify findings that can assist in improving the daily lives of the elderly.

The DSST quartile sensitivity test showed that the prevalence of the middle two groups was significantly lower than in the lowest quartile, and there was no significant correlation in the highest quartile. In CERAD-WL, CERAD-DR, and AFT sensitivity tests, the fully adjusted models showed no significant correlation (Supplemental Table 1). We also found a nonlinear relationship between the three sensitivity tests (Supplemental Figures 1, 2 and 3) with inflection points of 1.13, 1.93, and 2.09, respectively. These tests are biased differently in cognition. It is widely acknowledged that DSST is sensitive to examining processing speed, executive function, and working memory [41]. CERAD-WL and CERAD-DR examine memory, while AFT assesses language ability. These results further suggest that environmental lighting is associated with cognitive impairment.

We also analyzed the acceleration data obtained while wearing ActiGraph model GT3X and found that the sum of the triaxial acceleration of the elderly with cognitive injury was smaller. We calculated the interaction effects between the lux value and the MIMS triaxial value and found no significant interaction, suggesting that the contribution of light to cognitive function is not related to the amount of activity.

Limitations of the present study should be acknowledged. First, DSST was only conducted in specific year cycles, and the study population was relatively small. Furthermore, it is widely acknowledged that cross-sectional studies are limited, in that, they can only provide an association between environmental light and cognitive impairment, and a protective effect of environmental light on cognition could not be substantiated. Next, although several relevant confounding factors were adjusted, the influence of other confounding factors, such as seasonality and the effect of participants' socioeconomic status, could not be excluded. Finally, the NHANES database only contains data on the amount of ambient light and does not take into account other properties of light, such as wavelength and frequency.

## 5. Conclusion

In summary, our results suggested that the level of AI may be linked to cognitive impairment. We found a nonlinear relationship between AI and cognitive impairment. More prospective studies are needed to further investigate the role of AI in cognitive impairment.

## Figures and Tables

**Table 1:** Characteristics of the study population from NHANES 2011–2014 (*n* = 2,507).

Variable	Total	Normal cognition	Cognitive impairment	*P*-value
Age	69.01 (0.22)	68.40 (0.21)	72.92 (0.40)	<0.0001
Sex				
Female	1,282 (51.14)	1,019 (55.11)	263 (51.50)	0.28
Male	1,225 (48.86)	883 (44.89)	342 (48.50)	
Ethnicity				
Black	605 (24.13)	396 (6.38)	209 (21.36)	<0.0001
Hispanic	264 (10.53)	144 (2.31)	120 (13.20)	
Mexican American	218 (8.7)	140 (2.43)	78 (9.07)	
Other	226 (9.01)	188 (4.67)	38 (4.54)	
White	1,194 (47.63)	1,034 (84.21)	160 (51.83)	
Education				
Completed high school	591 (23.59)	456 (21.34)	135 (25.07)	<0.0001
Less than high school	611 (24.39)	268 (10.11)	343 (49.01)	
More than high school	1,303 (52.02)	1,178 (68.54)	125 (25.92)	
AI/100000, lux/100000	1.53 (0.07)	1.57 (0.07)	1.28 (0.09)	0.003
DSST	52.65 (0.63)	57.04 (0.55)	24.70 (0.40)	<0.0001
TG	124.81 (3.80)	124.41 (4.60)	127.20 (7.37)	0.78
HDL	55.52 (0.69)	55.97 (0.80)	52.56 (1.05)	0.02
eGFR	74.20 (0.41)	75.16 (0.45)	67.80 (1.28)	<0.0001
ACR	45.30 (4.68)	33.97 (3.99)	119.00 (24.52)	0.002
Sbp	130.92 (0.56)	129.93 (0.54)	137.49 (1.34)	<0.0001
Dbp	68.50 (0.44)	68.88 (0.49)	65.98 (0.68)	0.001
BMI	29.10 (0.19)	29.12 (0.20)	28.97 (0.38)	0.72
Waist	102.39 (0.45)	102.44 (0.50)	102.05 (1.04)	0.74
MIMS triaxial value	9481.47 (87.19)	9640.88 (92.43)	8583.05 (141.18)	<0.0001
CKD				
No	1,592 (66.61)	1,283 (72.94)	309 (48.94)	<0.0001
Yes	798 (33.39)	539 (27.06)	259 (51.06)	
MetS				
No	1,690 (67.41)	1,279 (66.58)	411 (68.77)	0.6
Yes	817 (32.59)	623 (33.42)	194 (31.23)	
Hypertension				
No	740 (29.52)	616 (35.95)	124 (19.21)	<0.0001
Yes	1,767 (70.48)	1,286 (64.05)	481 (80.79)	

Mean ± SD for continuous variables: *P*-value was calculated by one-way ANOVA. % for categorical variables: *P*-value was calculated by weighted chi-square test. MIMS: Monitor-Independent Movement Summary; CKD: chronic kidney disease; BMI: body mass index; AI: ambient illumination; DSST: digit symbol substitution test; MetS: metabolic syndrome; Sbp: systolic blood pressure; Dbp: diastolic blood pressure; TG: triglycerides; HDL: high-density lipoprotein. MIMS: Monitor-Independent Movement Summary; CKD: chronic kidney disease; BMI: body mass index; AI: ambient illumination; DSST: digit symbol substitution test; MetS: metabolic syndrome; Sbp: systolic blood pressure; Dbp: diastolic blood pressure; TG: triglycerides; HDL: high-density lipoprotein. Not all data were available for all participants.

**Table 2:** Association of AI and cognitive impairment.

	Crude model OR (95% CI)	*P*-value	Model 1 OR (95% CI)	*P*-value	Model 2 OR (95% CI)	*P*-value
Lux value	0.775 (0.647, 0.927)	0.007	0.882 (0.723, 1.077)	0.207	0.872 (0.699, 1.088)	0.211
Lux value × MIMS triaxial value	0.99 (0.95, 1.02)	0.47	0.99 (0.95, 1.04)	0.76	1.01 (0.97, 1.06)	0.56
Q1	Ref.		Ref.		Ref.	
Q2	0.558 (0.424, 0.736)	<0.001	0.565 (0.402, 0.796)	0.002	0.552 (0.358, 0.850)	0.01
Q3	0.502 (0.336, 0.751)	0.002	0.567 (0.377, 0.852)	0.008	0.536 (0.340, 0.847)	0.01
Q4	0.445 (0.291, 0.681)	<0.001	0.634 (0.377, 1.066)	0.083	0.620 (0.329, 1.166)	0.129
*P* for trend		0.001		0.104		0.129

Crude model, no covariate was adjusted; Model 1 was adjusted for age, gender, and race; Model 2 was adjusted for age, gender, race, the education level, and diagnosis of CKD, MetS, and hypertension.

**Table 3:** Estimation of change-points in the association between AI and cognitive impairment.

Outcome	OR (95% CI), *P*-value
Model 1 fitting model by standard linear regression	0.872 (0.699, 1.088), 0.211
Model 2 fitting model by two-piecewise linear regression	
Infection point	1.22
≤1.22	0.35 (0.18, 0.70), 0.005
>1.22	1.05 (0.71, 1.53), 0.81

Age, gender, race, the education level, and diagnosis of CKD, MetS, and hypertension were adjusted.

## Data Availability

This data can be found here: http://www.cdc.gov/nchs/nhanes/.

## Supplementary Materials

Supplementary materials Supplemental Figures 1, 2, and 3 and Supplemental Table 1 are the results of sensitivity analysis and smooth curve fitting.
