# Supplementary material for: Association between Ambient Illumination and Cognitive Impairment: A Population-Based Study of Older
Source: Behav Neurol. 2023 Apr 10;2023:4131377. doi: 10.1155/2023/4131377 (PMC10110376; doi:10.1155/2023/4131377)
Supplement: Supplementary materials — Supplemental Figures 1, 2, and 3 and Supplemental Table 1 are the results of sensitivity analysis and smooth curve fitting. [file 4131377.f1.zip › 4131377.f3.docx]

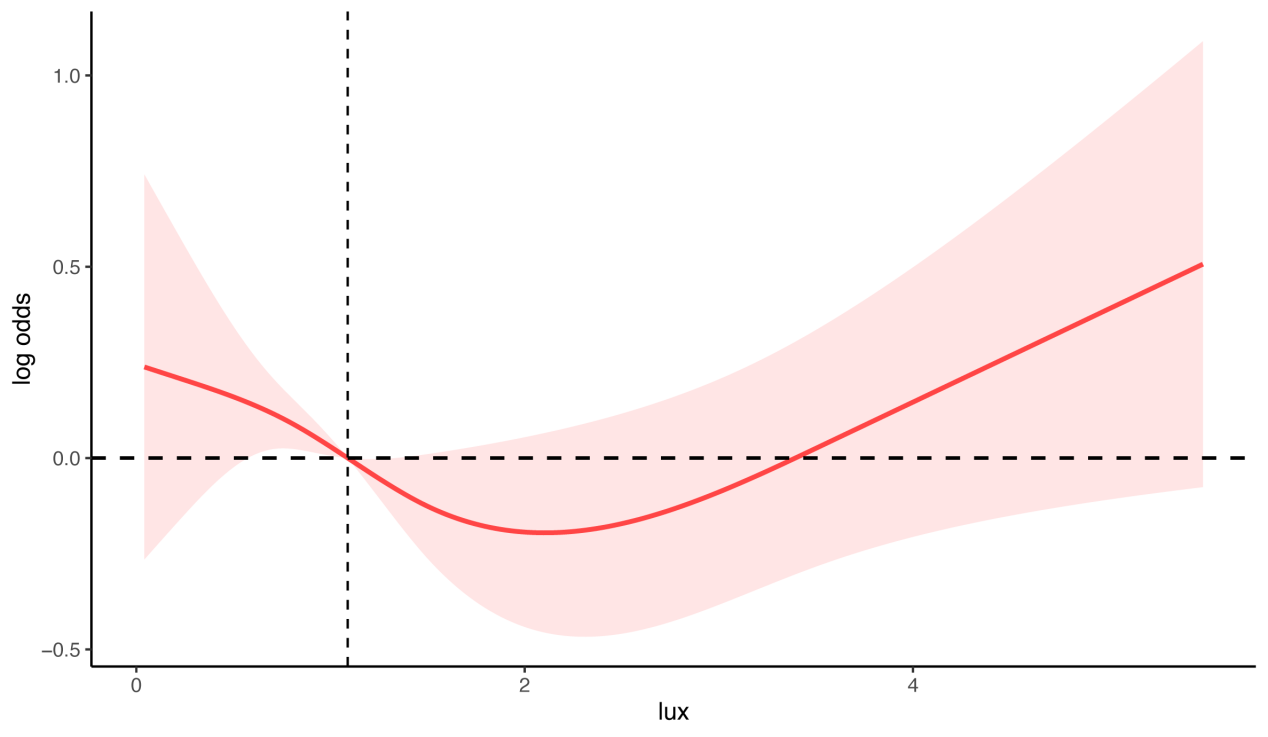


**Supplemental Figure 3.** Association between AI and cognitive impairment using AFT. Age, gender, and race, the education level, and diagnosis of CKD, MetS, hypertension were adjusted.
