# Supplementary material for: Association between Ambient Illumination and Cognitive Impairment: A Population-Based Study of Older
Source: Behav Neurol. 2023 Apr 10;2023:4131377. doi: 10.1155/2023/4131377 (PMC10110376; doi:10.1155/2023/4131377)
Supplement: Supplementary materials — Supplemental Figures 1, 2, and 3 and Supplemental Table 1 are the results of sensitivity analysis and smooth curve fitting. [file 4131377.f1.zip › 4131377.f4.docx]

**Supplemental Table 1.** Association of AI and cognitive impairment.

|  | Crude model OR (95% CI) | P-value | Model 1 OR (95% CI) | P-value | Model 2 OR (95% CI) | P-value |
| --- | --- | --- | --- | --- | --- | --- |
| WL | 0.886(0.778,1.009) | 0.066 | 0.940(0.805,1.099) | 0.424 | 0.952(0.810,1.119) | 0.532 |
| DR | 0.907(0.771,1.067) | 0.23 | 0.953(0.798,1.138) | 0.583 | 0.965(0.814,1.145) | 0.67 |
| AFT | 0.854(0.769,0.948) | 0.004 | 0.972(0.867,1.091) | 0.62 | 0.982(0.871,1.107) | 0.76 |

Crude model, no covariate was adjusted;

Model 1 was adjusted for age, gender, and race;

Model 2 was adjusted for age, gender, and race, the education level, and diagnosis of CKD, MetS, hypertension.
